# Improvement in creativity after transcranial random noise stimulation (tRNS) over the left dorsolateral prefrontal cortex

**DOI:** 10.1038/s41598-019-43626-4

**Published:** 2019-05-08

**Authors:** Javier Peña, Agurne Sampedro, Naroa Ibarretxe-Bilbao, Leire Zubiaurre-Elorza, Natalia Ojeda

**Affiliations:** Department of Methods and Experimental Psychology, Faculty of Psychology and Education, University of Deusto, Bilbao, Basque Country Spain

**Keywords:** Neuroscience, Neurology

## Abstract

Creativity has previously been shown to improve after the application of direct and alternating current transcranial stimulation over the dorsolateral prefrontal cortex (DLPFC). However, previous studies have not tested whether transcranial random noise stimulation (tRNS) was efficient for this purpose. The aim of this randomized, double-blind, placebo-controlled study was to investigate the effect of tRNS on both verbal convergent and (verbal and visual) divergent thinking during left DLPFC tRNS stimulation. Thirty healthy participants were randomly allocated to either a tRNS active group or a sham group. Each session lasted 20 min and the current was set to 1.5 mA (100–500 Hz). Participants’ verbal convergent thinking was assessed with the Remote Associates Test (RAT). Verbal and visual divergent thinking were respectively measured by using the Unusual Uses and Picture Completion subtests from the Torrance Tests of Creative Thinking. Bootstrapped analysis of variance showed significant differences in the mean change scores between the active tRNS group and the sham group in RAT scores (*d* = 1.68); unusual uses: fluency (*d* = 2.29) and originality (*d* = 1.43); and general creativity (*d* = 1.45). Visual divergent thinking, in contrast, did not show any significant improvement. Our results suggested that tRNS over the left DLPFC is effective for increasing verbal divergent and convergent thinking.

## Introduction

Creativity plays a key role in many areas of human life, as has been previously suggested^1^. An increasing number of studies have attempted to investigate the neurological basis for creativity using either MRI^2,3^ or EEG techniques^4^. Additional evidence of brain areas and networks related to creativity performance has come from studies that included noninvasive transcranial stimulation A review recently carried out by Weinberger *et al*.^5^ indicated that creative cognition may involve two main processes: idea generation (which depends on the availability of unfiltered information), and idea selection (which includes task-directed thoughts and integration of semantically distant concepts). Each of these processes has been suggested to rely primarily on different neural mechanisms^6^.

More specifically, idea generation has been mostly related to cathodal Transcranial Direct Current Stimulation (tDCS) of the left inferior frontotemporal cortex, including the anterior temporal lobe^7,8^, inferior frontal gyrus^9^ and prefrontal cortex^10^. This improvement in idea generation generally refers to divergent thinking. Divergent thinking has been defined as the ability to simultaneously establish remote associations between unrelated concepts from distant categories, as well as to generate multiple alternative and novel answers to a single problem^11^. For example, Chrysikou *et al*.^10^ reported a higher number of uncommon uses for everyday objects (verbal divergent thinking) after inhibiting the left prefrontal cortex. Similarly, Mayseless and Shamay-Tsoory^9^ found that cathodal stimulation of the left inferior frontal gyrus improved the performance in the Alternate Uses Task (verbal divergent test)^12^. More specifically, it improved fluency and flexibility, but not originality. Chi and Snyder^7,8^ found a higher solution rate for very challenging insight problems (matchstick arithmetic and the nine dot problem) after inhibiting the left anterior temporal lobe. They hypothesized that the higher solution rate for both problems was due to the induced ability to consider novel approaches, instead of stored mental templates and contexts through the inhibition of the left anterior temporal lobe.

Idea selection has been more closely related to the dorsolateral prefrontal cortex (DLPFC). For example, Cerruti *et al*.^13^ found a significant improvement in the Remote Associates Test (RAT)^14^ after anodal stimulation over the left DLPFC compared to both cathodal and sham conditions. The RAT consists of three words that appear to be unrelated. Participants have to find a word that can be linked simultaneously to those three words^14^. The RAT has been considered to be a measure of verbal convergent thinking, which involves finding a single solution to a problem in a deductive way^15^. This test is supposed to contain both a generative component (since participants are asked to produce a remotely related word), and selectivity (participants must select a correct solution among the possible words generated)^5^. Additional evidence of verbal convergent thinking improvement after anodal stimulation over the left DLPFC came from the study carried out by Zmigrod *et al*.^15^, who reported significantly better performance in a similar version of the RAT (Compound Remote Associates Task)^16^. Colombo *et al*.^17^ additionally found a significant improvement in verbal divergent thinking (alternate uses task), but only after priming on creative thinking. This last finding suggests that stimulation of the left DLPFC may also induce higher divergent thinking scores by asking participants to deliberately search for more unusual answers. This additional cognitive demand may increase the need for selectivity^5^, which may be a reason for being positively affected by left DLPFC stimulation.

Another recent study^18^ found an increase in insight problems (matchstick arithmetic problems) after cathodal (instead of anodal) stimulation of the left DLPFC. This measure is mainly based on constraint relaxation, whereas verbal convergent thinking (RAT) and divergent thinking (Alternate Uses Task) additionally require high-level selectivity processes.

All the above-mentioned studies used tDCS. This kind of transcranial stimulation increases the excitability of the area under positively charged anodal electrode, and decreases the excitability of the area under the cathodal electrode^19^.

More recently, some researchers^20,21^ introduced a different type of transcranial stimulation in creative enhancement research, namely Transcranial Alternating Current Stimulation (tACS). These two studies reported an increase in creative skills following 10 hZ tACS stimulation of DLPFC. More specifically, Lustemberg *et al*.^20^ evaluated the effect of 10 Hz tACS on visual divergent creativity (Torrance Test of creative Thinking), and found that the tACS group significantly improved their general creativity index, but none of the subscores (fluency, originality, elaboration, abstract of titles and resistance to premature closures). Grabner *et al*.^21^ used a similar montage and assessed verbal divergent creativity (alternate uses task). They found a significant improvement in ideational fluency, but not in originality.

Transcranial Random Noise Stimulation (tRNS) recently emerged as a promising alternative to tDCS and tACS. tRNS is a form of transcranial electrical stimulation of random noise to modulate cortical plasticity^22^. The underlying mechanisms for this cortical excitability changes are not yet fully understood.

One of the proposed mechanisms of tRNS is the increase of neuronal excitability via stochastic resonance, whereby weak neural signal detection in the central nervous system is enhanced when ‘noise’ is added^23^. An additional potential mechanism may be related to the repetitive opening of NaC channels^24^.

Regarding the effectiveness of tRNS, some studies have suggested that this stimulation may be more pronounced than tDCS in visual perceptual learning tasks^22^, and that the effect may also last longer^25^. Moreover, tRNS seems to be more tolerable than tDCS with respect to irritation and burning^26^. For example, Inukai *et al*.^27^ compared tACS, tDCS and tRNS for increasing cortical excitability and reported that tRNS resulted in the most significant increase. Although not directly related to cognitive performance, Vanneste *et al*.^28^ directly compared the response of patients with tinnitus after using tDCS, tACS and tRNS techniques, and showed that tRNS was superior to both tDCS and tACS. However, there was also evidence of a lack of positive tRNS effects in numerical cognition after parietal tRNS^29^. tRNS has also been used among children with learning disabilities^30^, where tRNS over the bilateral DLPFC was found to improve learning and performance. Additional evidence of the difference between tDCS and tRNS has come from Antal *et al*.’s study^31^. These authors compared the effect of the brain-derived neurotrophic factor (BDNF) gene on the response to both tDCS and tRNS among healthy people. Their findings suggested that tDCS was more effective for carriers of the Val66-Met allele, whereas tRNS produced similar effects on both Val/Val and Val/Met carriers. A recent meta-analysis^32^ indicated that the overall effect of tRNS on language and mathematics was stronger than that of tDCS. However, as the study claimed^32^, the low number of investigations using tRNS prevented the authors from drawing firm conclusions. Altogether, evidence has suggested that tRNS is more effective than tDCS regarding some cognitive skills (such as learning, and language or numerical cognition), and that it can be extrapolated to participants regardless of at least some genetics (BDNF). However, as far as the authors are aware, none of these previous studies have attempted to assess if left DLPFC tRNS improves creative performance. Lustenberger *et al*.^20^ suggested that tests should be conducted to explore if tRNS would also enhance creative thinking.

Therefore, the goal of the current study was to test if tRNS over the left DLPFC improves different domains of creativity, including convergent thinking and both verbal and visual divergent thinking.

## Results

### Baseline characteristics of active tRNS and sham groups

Table 1 shows the baseline characteristics of both groups. There were no significant differences in age (*p* = 0.32), gender (*p* = 0.27), years of education (*p* = 0.91), Edinburgh Handedness Inventory (*p* = 0.29), Idea Evaluation Self-Efficacy (*p* = 0.78) or semantic fluency (*p* = 0.81). Number of hours slept the previous night were also equal (*p* = 0.68). Participants were asked if the number of hours slept and number of stimulant drinks ingested were more than usual, less than usual, or as usual. The were no significant differences between the groups in the proportion of these responses regarding sleeping condition (*p* = 0.33). The number of stimulant drinks (tea, coffee, or similar) ingested was also equivalent (*p* = 0.46), as well as the proportion of less than usual, more than usual, or as usual responses (*p* = 0.51).

**Table 1:** Participant characteristics at baseline.

	Active tRNS Group (n = 15)		Sham Group (n = 15)		*p*
	Mean (95% CI)	SD	Mean (95% CI)	SD	
Age (years)	31.2 (27.6 to 34.8)	6.4	34.3 (28.8 to 39.9)	10.0	0.32
Years of education (years)	16.8 (15.5 to 18.1)	2.3	16.7 (14.9 to 18.4)	3.2	0.91
Gender: n (%)					
Males	9 (60.0%)		6 (40.0%)		0.27
Verbal Fluency	25.3 (21.6 to 28.9)	6.3	25.8 (22.3 to 29.4)	5.9	0.81
Idea Evaluation Self-Efficacy	30.6 (27.7 to 33.5)	4.8	30.1 (27.9 to 32.4)	4.0	0.78
Number of hours slept	6.8 (6.0 to 7.5)	1.2	6.9 (6.3 to 7.5)	1.1	0.68
Number of stimulants taken	1.0 (0.3 to 1.7)	1.1	1.3 (0.8 to 1.8)	0.8	0.46
Edinburgh Handedness Inventory	79.2 (67.3 to 91.1)	21.5	86.3 (75.5 to 97.2)	19.6	0.35

Abbreviations: tRNS = Transcranial Random Noise Stimulation; CI = Confidence Interval; SD = Standard Deviation.

Table 2 shows the mean and SD of the creativity scores from both groups at baseline and after tRNS (active or sham). There were no significant differences between the groups in any of the creativity scores at baseline, including RAT (*F* = 2.79, *p* = 0.11), UU fluency (*F* = 0.06, *p* = 0.80), UU originality (*F* = 0.05, *p* = 0.83), UU flexibility (*F* = 0.16, *p* = 0.70), figural creativity fluency (*F* = 0.58, *p* = 0.46), figural creativity originality (*F* = 0.01, *p* = 0.91), figural creativity elaboration (*F* = 1.01, *p* = 0.32) and total creativity score (*F* = 0.65, *p* = 0.43).

**Table 2:** Creativity scores in the active tRNS and the sham group at Pre- and post-intervention.

		Active tRNS Group	SD	Sham Group	SD
		Mean (95% CI)		Mean (95% CI)	
RAT	Pre	11.3 (9.6 to 13.1)	3.3	13.3 (12.6 to 16.4)	3.2
	Post	14.5 (12.2 to 16.6)	3.2	12.5 (10.7 to 14.4)	3.9
Figural fluency	Pre	5.1 (3.8 to 6.4)	2.4	6.0 (4.7 to 7.3)	2.3
	Post	7.0 (5.7 to 8.3)	2.4	6.6 (5.3 to 7.9)	2.4
Figural Originality	Pre	2.5 (1.4 to 3.6)	1.9	2.6 (1.5 to 3.7)	2.3
	Post	2.5 (1.4 to 3.6)	2.9	2.1 (1.1 to 3.2)	1.7
Figural Elaboration	Pre	15.1 (9.9 to 20.3)	7.1	19.5 (14.2 to 24.7)	12.0
	Post	21.2 (17.4 to 25.0)	7.1	18.0 (14.2 to 21.8)	7.3
UU fluency	Pre	8.6 (7.0 to 10.2)	2.9	8.9 (7.3 to 10.5)	3.1
	Post	11.3 (9.8 to 12.7)	2.9	8.4 (7.0 to 12.7)	2.5
UU Originality	Pre	5.7 (4.3 to 7.1)	2.3	6.1 (4.7 to 7.5)	2.8
	Post	9.0 (7.6 to 10.3)	2.6	6.1 (4.7 to 7.5)	2.5
UU Flexibility	Pre	7.1 (6.0 to 8.2)	2.4	6.9 (5.8 to 8.0)	1.6
	Post	7.8 (6.6 to 8.9)	2.3	6.8 (5.6 to 8.0)	2.1
General creativity	Pre	−0.1 (−0.5 to 0.3)	0.7	0.1 (−0.2 to 0.3)	0.6
	Post	0.3 (−0.13 to 0.7)	0.7	−0.3 (−0.63 to 0.10)	0.7

Abbreviations: tRNS = Transcranial Random Noise Stimulation; CI = Confidence Interval; SD = Standard Deviation; RAT = Remote Associates Test; UU = Unusual Uses Test from The Torrance Test of Creative Thinking.

### Effect of tRNS on creativity

Regarding change scores (see Table 3), results showed that the active tRNS group improved significantly when compared to the sham condition group in RAT, showing a very large effect size (Cohen’s *d* = 1.68).

**Table 3:** Differences in change scores between the active tRNS group and the sham group.

	Active tRNS Group		Sham group		ANOVA for change scores		Effect size
	Mean Change Score (95% CI)	SD	Mean Change Score (95% CI)	SD	F	*p* ^a^	*d* (95% CI)
RAT	3.90 (2.3 to 5.7)	3.01	−1.06 (−2.5 to 0.4)	2. 89	18.14	<0.001	1.68 (0.81 to 2.46)
Figural fluency	1.63 (0.5 to 2.8)	1.96	0.60 (−0.3 to 1.6)	2.02	1.70	0.20	0.50 (−0.24 to 1.22)
Figural originality	0.18 (−1.0 to 1.4)	2.08	−0.46 (−1.4 to 0.4)	1.72	0.75	0.40	0.35 (−0.38 to 1.06)
Figural elaboration	6.07 (1.0 to 11.2)	11.84	−1.46 (−6.2 to 3.2)	9.99	4.17	0.05	0.68 (−0.07 to1.40)
UU fluency	3.54 (2.1 to 5.2)	2.54	−0.46 (−1.5 to 0.5)	1.75	20.69	<0.001	2.29 (1.32 to 3.14)
UU originality	3.27 (2.3 to 4.1)	1.81	−0.07 (−1.4 to 1.3)	2.76	15.28	<0.001	1.43 (0.60 to 2.19)
UU Flexibility	0.69 (−0.6 to 2.1)	2.69	−0.06 (−1.0 to 0.9)	1.75	0.83	0.37	0.31 (−0.42 to 1.02)
Total creativity	0.37 (0.1 to 0.6)	0.52	−0.37 (−0.6 to −0.1)	0.50	15.43	0.001	1.45 (0.61 to 2.21)

Abbreviations: tRNS = Transcranial Random Noise Stimulation; CI = Confidence Interval; SD = Standard deviation; RAT = Remote Associates Test; UU = Unusual Uses Test from The Torrance Test of Creative Thinking; Change score = post intervention score minus baseline score; *d* = Cohen’s d. CI and Standard Errors (SEs) from bootstrap analyses.

^a^Significance levels were determined using F tests based on the estimated SEs from bootstrap analyses for that comparison, rather than using a pooled estimate to show SEs.

The results regarding unusual uses also showed a significant improvement in scores related to fluency (Cohen’s *d* = 2.29) and originality (Cohen’s *d* = 1.43), but not to flexibility.

Figural creativity, in contrast, did not show any significant improvement after tRNS when compared to the sham group. Only figural elaboration showed a marginally significant effect (*p* = 0.051).

Finally, total creativity significantly improved after tRNS when compared to the sham group, showing a large effect size (Cohen’s *d* = 1.45).

### Adverse effects questionnaire

Participants reported having no discomfort or unusual sensation on their scalp. None of the participants reported having experienced any significant adverse effects. There were no significant differences between the groups in terms of the number of adverse effects (*F* = 0.24, *p* = 0.63). However, a closer inspection of phosphenes revealed that the active tRNS group reported having experienced this effect more often than the sham group (*F* = 4.66, *p* = 0.04).

## Discussion

The objective of this study was to test if a new kind of noninvasive transcranial stimulation, Random Noise Stimulation (tRNS), could be effective in improving creative cognition in healthy people. Results confirmed that participants receiving real tRNS over the Left Dorsolateral Prefrontal Cortex (DLPFC) improved both verbal convergent thinking (RAT scores) and verbal divergent thinking (UU), but not visual divergent thinking. More specifically, fluency and originality scores from verbal divergent thinking significantly improved after receiving tRNS. The effect size was very large for all of them (Cohen’s *d* ranged from 1.43 to 2.29).

As far as the authors are aware, none of the previous studies have tested if tRNS improves creativity, so a direct comparison is not possible. However, these results are in line with previous studies that used tDCS over the left DLPFC^13,15^. More specifically, Cerruti *et al*.^13^ found a significant improvement in verbal convergent thinking (RAT scores) after anodal stimulation over the left DLPFC. Based on their reported *t* score [Cohen’s *d* = 2*t*/√(*df*)], the effect size for RAT was 1.63, almost identical to our results. Similarly, Zmigrod *et al*.^15^ found significant improvement after anodal tDCS over the left DLPFC in a similar version of RAT (Compound Remote Associates Task)^16^. Their results regarding alternate uses did not reach statistically significant results, but scores were systematically higher for fluency, flexibility and elaboration, which is consistent with our results.

Results from studies that used tACS and divergent thinking (Alternate Uses task)^21^ were also partially consistent with our results, since their positive effects were circumscribed only to fluency scores, whereas tRNS also produced a significant and large improvement in originality. The improvement in originality is highly remarkable, given that most studies addressing DLPFC affected mainly fluency in divergent thinking, but not originality^15,20,21^. As far as the authors are aware, only one study has demonstrated a significant improvement in originality after anodal stimulation over the left DLPFC^17^. More specifically, these authors showed that a constant current of 1.5 mA for 20 minutes (anode stimulation over left DLPFC and cathode stimulation over ipsilateral mastoid) was enough to significantly improve originality scores in verbal divergent thinking (the Product Improvement subtest from the Torrance Test of Creative Thinking), when combined with divergent priming based on mental stimulation task. The authors^17^ explained this divergent priming as a way of inducing people to adopt a particular attitude towards a creative task by visualizing themselves using an object in an unusual way. In other words, a similar creative output might be reached through both the inhibition of left inferior frontotemporal cortex (involved in inhibitory control and retention of previous experiences)^5^ and the stimulation over the left DLPFC (by enhancing one’s thoughts towards a specific aim under an increase in selection demands).

Visual divergent thinking, however, did not see a significant improvement after tRNS stimulation in any of the dimensions assessed (fluency, originality, and elaboration). Only figural elaboration was marginally significant, indicating that tRNS may partially enhance visual based divergent thinking. Contrary to our results, Lustenberger *et al*.^20^ found a significant improvement in general figural creativity after tACS over the DLPFC. One possible reason for the lack of significant improvement in visual divergent thinking may be that left side stimulation especially enhanced verbal-based tasks. Most previous studies have not included similar kinds of visual creative tasks, so future research is needed in order to clarify if other brain networks are more related to visual divergent thinking or to a more visual-based creative outcome, such as painting or architecture.

Research on improvement in creativity after transcranial stimulation has also been focused on other brain areas, such as the anterior temporal lobe^7,33,34^, prefrontal cortex^10^, inferior frontal gyrus^9,35^ and posterior parietal cortex^36,37^. Some studies have reported increased verbal divergent thinking (Alternate Uses Task) after cathodal stimulation over the left inferior frontal gyrus^9,35^. Contradictory results have been found regarding insight type problem solving (matchstick arithmetic), as one study reported a significant increase after cathodal stimulation over the left after anterior temporal lobe^7^, whereas another study^34^ found results that were not significant. Regarding verbal convergent thinking (RAT), inconclusive results have been reported so far. For example, Ruggiero *et al*.^33^ and Aihara *et al*.^34^ found no significant changes in RAT scores after both anodal and cathodal stimulation over the anterior temporal lobe. Results from posterior parietal cortex stimulation^15^ on RAT total scores showed no significant improvement. However, both anodal left-cathodal right and cathodal left-anodal right stimulation over the posterior parietal cortex showed a significant increase in the number of solutions to insight type problems in the RAT when compared to the sham condition. Altogether, results regarding verbal convergent thinking (RAT) suggest that better performance in this test depends more on the stimulation over the left DLPFC. This idea is consistent with the larger evidence of improvement after left DLPFC stimulation when compared to the involvement of other areas, such as the anterior temporal lobe^34^ or the posterior parietal cortex^15^ (although these authors found a higher rate of solutions to insight type solutions in the RAT after bilateral stimulation over the posterior parietal cortex).

During the study, a very interesting situation was encountered that may be a case of serendipity. A participant came back to the lab about ten minutes after finishing the experiment and reported having answered an email on his smartphone that involved a highly demanding cognitive task. He considered himself really bad at remembering names and the email asked him to recall a list of about twenty names and surnames of students that he reported he had been unable to remember the day before. Surprisingly for him, he was able to recall all of them without looking at the list. After finishing the experiment, we confirmed that he had received real tRNS. This interesting case may provide some indication about the potential effect that this specific montage and stimulation (tRNS) has on memory. Future studies could investigate if this was only an isolated case, or if it has a real effect on human memory.

There are several limitations to this study. Firstly, the time provided for verbal and figural creativity tests was limited to 2 minutes for each subtest from the TTCT. Although direct comparison with other standard studies was not possible, as we were more focused on change scores in creative tests after transcranial stimulation than on the participants’ creative profile compared to the general population. Another limitation is that we did not assess the cathodal stimulation over the left DLPFC, so direct comparison of both stimulations was not available.

The results derived from the current study raise some questions that could be addressed in future studies. Given the large effect sizes found in the RAT and UU, it would be interesting to directly compare three kinds of stimulation (tACS, tDCS and tRNS) over the same brain region (left DLPFC) and assess if one of them is more effective and tolerable than the others. Related to the last point, future lines of research should also test the different pattern of effect that several montages (including left DLPFC and inferior frontotemporal cortex) could have on different aspects of creative thinking (including creative idea generation and creative idea selection, or visual vs verbal divergent thinking). High definition transcranial stimulation would also be helpful to disentangle the differential effect of close areas on different aspects of creative thinking. Finally, it would be also interesting to investigate the baseline characteristics related to a better response on creative cognition after transcranial stimulation, such as age, gender, previous level of creative abilities and, as suggested by Antal *et al*.^31^, the effect of genes that have an influence on human brain functions. More research focusing on these factors may help explain why some healthy people or participants with neurological conditions do not benefit from these brain stimulation techniques.

## Method

### Participants

Thirty healthy participants were recruited. Inclusion criteria included both genders and being 18 years old or above. Exclusion criteria included the following: (1) previous history of brain surgery; (2) previous history or presence of a neurological disorder or a neurological injury (epileptic or convulsive seizure, brain stroke, severe brain injury); (3) suffering from frequent or severe headaches, including migraines; (4) presence of some kind of metallic implant in the brain (outside of the mouth); and (5) being pregnant.

Participants did not receive any kind of financial compensation. Ethical approval was obtained from the Deusto University Ethics Committee (Ref: ETK-31/17-18).

All participants were volunteers and gave written informed consent prior to their participation in the study, in accordance with the tenets of the Declaration of Helsinki. They were free to withdraw from the study at any time. Following ethical aspects, the sham group was offered active tRNS once the study was finished and the group condition had been revealed.

### Measures

#### The Edinburgh Handedness Inventory

Previous research has demonstrated that handedness is to some extent related to creativity^38^, so the Edinburgh Handedness Inventory^39^ was used to assess handedness. This is a self-reported questionnaire where participants were asked to indicate their preference of hand use for 10 everyday activities. Possible responses for each activity were no preference (0 points), preference (1 point), and very strong preference (2 points). In order to assess handedness, a consistency formula (right − left/right + Ieft) was used. Scores ranged from 100 (perfectly right-handed) to −100 (perfectly left-handed).

#### Calibrated Ideational Fluency Assessment

A verbal fluency task was included in order to control for possible baseline differences in verbal fluency between active and sham groups, since previous research led us to expect to find improvement in fluency scores for divergent thinking^10,17^. Semantic Fluency was measured with the Calibrated Ideational Fluency Assessment (CIFA)^40^. A category-cued task was used where participants were asked to report words related to the semantic category of animals for one minute. The total number of correct responses was recorded.

#### Idea Evaluation Self-Efficacy

Some previous studies^21,41^ have suggested that higher baseline creative potential may affect the effectiveness of brain stimulation. Therefore, the Spanish version of the Idea Evaluation Self-Efficacy^42^ was included to assess perceived ability to evaluate original ideas. This scale consisted of 8 items, and responses ranged from 1 (strongly disagree) to 5 points (strongly agree). The internal consistency of this scale was very high (Cronbach’s alpha = 0.84). Higher scores reflected higher self-efficacy for evaluation of ideas.

#### The Torrance Test of Creative Thinking (TTCT)

The Torrance Test of Creative Thinking (TTCT) is one of the most widely used instruments for the assessment of divergent thinking^43^. Picture Completion and the Unusual Uses (UU) subtests from the TTCT^44^ were administered in order to assess visual divergent thinking and verbal divergent thinking, respectively. Each task had to be performed in two minutes. Different forms of the test (Form A and Form B) were administered for the Pre- and the Post-intervention. The Picture Completion task requires participants to complete ten unfinished figures with additional elements. Although the original test includes many dimensions, only three dimensions were measured for this study: fluency, originality, and elaboration. Fluency referred to the total number of relevant responses. The originality score was based on the statistical infrequency of each response. Elaboration was defined as the number of details added to the minimum basic response to the stimulus. In the Unusual Uses task, participants were asked to write as many unusual uses as possible for an item. In Form A of the test, the stimulus for which participants had to write unusual uses was Cardboard Boxes, while in Form B Tin Cans was used as a stimulus. Three dimensions were measured: fluency, originality, and flexibility. Fluency was based upon the number of different unusual uses produced. Originality was based on the statistical unusualness of each response. The flexibility score was obtained from the number of different categories represented in the responses.

#### The Remote Associates Test (RAT)

The Spanish version of the RAT (Mednick, 1962; Mednick & Mednick, 1967) was administered to assess verbal convergent thinking. This activity consists in identifying a solution word that is associated with three cue words. The solution can be related to the three cue words either semantically or by forming a compound word. Different forms of the test were employed for the Pre- and Post- intervention. Each form included 30 items. Participants were given 15 seconds for each item and the whole task lasted 7 minutes and 30 seconds. The internal consistency for the total score was very high (Cronbach’s alpha = 0.81).

#### Questionnaire of adverse effects

After each session, subjects filled out an 11-item questionnaire to assess any perceived side effects (e.g. headache, itching sensation, difficulty concentrating, phosphenes, etc.).

### tRNS protocol

**Figure 1:**
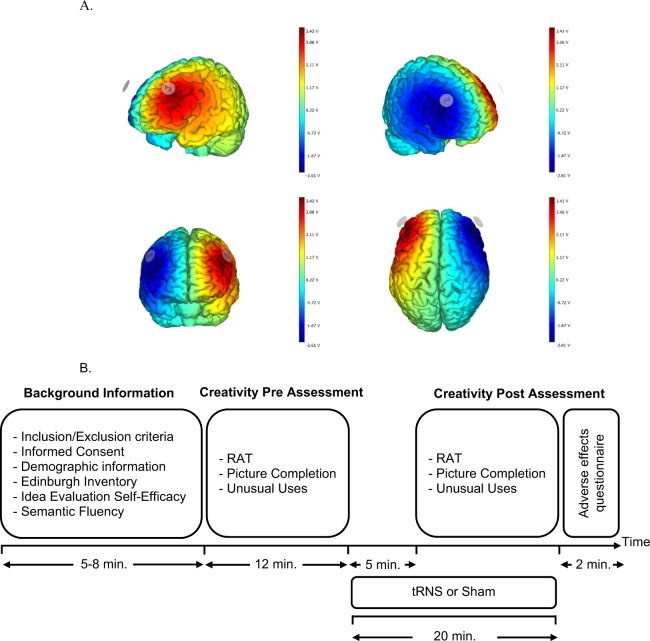
(**A**) Electrode Montages. Stimulation of the electric field according to *Stim Weaver* (Neuroelectrics, Spain) software based on a realistic head model derived from the Finite Element Method^33^. Red-yellow colors indicate increased magnitude of the total electric field due to tRNS. (**B**) Schematic representation of the experimental procedure (RAT = Remote Associates Test).

Participants in the active tRNS group received 1.5 mA of tRNS (100–500 Hz) to their bilateral dorsolateral prefrontal cortices (DLPFCs) via two saline-soaked (5 ml per sponge), 16 cm2 (8 × 8 cm^2^) circular sponges. They were attached under designated electrode positions (F3, F4) using a wireless tRNS cap that followed the International 10–20 system. Figure 1 shows the simulated electric field of this montage (based on Stim Weaver) using the finite element model^45^. tRNS was applied with a light, battery-operated device (Neuroelectrics Inc., Barcelona) attached to the back of the neoprene cap, delivering electrical current for 20 minutes, with additional ramp-up and ramp-down phases of 30 seconds. In the sham condition, current was applied for 30 seconds (with additional ramp-up and ramp-down phases of 30 seconds). The impedance of both electrodes was checked before and during tRNS application to ensure that it was under 10 kΩ.

### Procedure

An *a priori* power analysis was conducted to determine the sample size based on a previous study^20^ using G Power 3 software^46^. A sample size of 28 subjects, 14 in each group, was enough to attain an effect size (Cohen’s *d*) of 0.56 to detect between-group differences in creativity scores, with 80% power and 5% level of significance. The study had a double-blind, sham-controlled, parallel-group design. Recruitment and enrollment took place between June 2018 and November 2018. Afterwards, the participants were randomly allocated to either the active tRNS group or the sham group (see Fig. 1). Allocation to each group was conducted based on a computer-generated randomization of the list of participants (randomizer.com). All raters (JP and AS) were blind to treatment condition.

Figure 1 shows the design and procedure used in this study. After signing the consent form, participants provided sociodemographic information, along with hours of sleep and stimulant drinks ingested before the session. The exact time of assessment and stimulation was also recorded for each participant. Afterwards, Edinburgh Handedness Inventory, Idea Evaluation Self-Efficacy and semantic fluency tests were administered. A baseline creativity assessment was carried out before starting tRNS stimulation (active or sham). They have 7 minutes 30 seconds for RAT, 2 minutes for UU and 2 minutes for Picture completion. Five minutes after stimulation started, the parallel versions of the RAT, UU and Figure completion tests were performed with the same time limitations. The order of the versions of the RAT, UU and Figure completion tests were counterbalanced in order to control for “order” effects. Finally, participants completed the adverse effects questionnaire.

### Statistical analyses

Given the sample size (n < 50)^47^, normality of data was tested using the Shapiro-Wilk test. All variables appeared as normal distributions. Categorical data were analyzed with the x2 test. Baseline characteristics were compared using ANOVA. In order to analyze a more general creativity performance, a composite score was created. All creativity scores (RAT, UU and Figural creativity) were converted into Z-scores based on the pooled group. The total creativity composite score showed high internal consistency (Cronbach’s alpha = 0.80).

Change scores (post intervention minus baseline) between the active tRNS group and the sham group were compared on each of variables with an ANOVA. To obtain adjusted mean differences in change scores, we used bootstrapping^48^, a resampling technique in which random subsamples are generated from the observed sample. We generated 1,000 subsamples from within each group (with replacement).

Effect size (Cohen’s *d* and 95% confidence interval) was calculated based on change score differences between groups. IBM SPSS version 23.0^49^ was used for all statistical analyses. The significance level was set at 0.05. All tests were two-tailed.

## Data Availability

The datasets generated during and/or analyzed during the current study are available from the corresponding author on reasonable request.

## References

[1] Liu, Z. *et al*. Neural and genetic determinants of creativity. *Neuroimage*, 10.1016/j.neuroimage.2018.02.067 (2018). 10.1016/j.neuroimage.2018.02.067 29518564

[2] Jung, R. E. & Vartanian, O. *The Cambridge Handbook of the Neuroscience of Creativity*, 10.1017/9781316556238 (Cambridge University Press., 2018).

[3] Gonen-Yaacovi, G. *et al*. Rostral and caudal prefrontal contribution to creativity: a meta-analysis of functional imaging data. *Front. Hum. Neurosci*., 10.3389/fnhum.2013.00465 (2013). 10.3389/fnhum.2013.00465 PMC3743130 23966927

[4] Fink, A. & Benedek, M. EEG alpha power and creative ideation. *Neuroscience and Biobehavioral Reviews*, 10.1016/j.neubiorev.2012.12.002 (2014). 10.1016/j.neubiorev.2012.12.002 PMC4020761 23246442

[5] Weinberger, A. B., Green, A. E. & Chrysikou, E. G. Using Transcranial Direct Current Stimulation to Enhance Creative Cognition: Interactions between Task, Polarity, and Stimulation Site. *Front. Hum. Neurosci*., 10.3389/fnhum.2017.00246 (2017). 10.3389/fnhum.2017.00246 PMC5432551 28559804

[6] Beaty, R. E., Benedek, M., Kaufman, S. B. & Silvia, P. J. Default and Executive Network Coupling Supports Creative Idea Production. *Nat. Publ. Gr*. 1–14, 10.1038/srep10964 10.1038/srep10964 PMC4472024 26084037

[7] Chi, R. P. & Snyder, A. W. Facilitate insight by non-invasive brain stimulation. *PLoS One*, 10.1371/journal.pone.0016655 (2011). 10.1371/journal.pone.0016655 PMC3032738 21311746

[8] Chi, R. P. & Snyder, A. W. Brain stimulation enables the solution of an inherently difficult problem. *Neurosci. Lett*., 10.1016/j.neulet.2012.03.012 (2012). 10.1016/j.neulet.2012.03.012 22440856

[9] Mayseless, N. & Shamay-Tsoory, S. G. Enhancing verbal creativity: Modulating creativity by altering the balance between right and left inferior frontal gyrus with tDCS. *Neurosciencero*, 10.1016/j.neuroscience.2015.01.061 (2015). 10.1016/j.neuroscience.2015.01.061 25659343

[10] Chrysikou, E. G. *et al*. Noninvasive transcranial direct current stimulation over the left prefrontal cortex facilitates cognitive flexibility in tool use. *Cogn. Neurosci*., 10.1080/17588928.2013.768221 (2013). 10.1080/17588928.2013.768221 PMC3719984 23894253

[11] Guilford, J. P. *The nature of human intelligence*. *McGraw Hill*, 10.4018/978-1-59904-426-2.ch007(1967).

[12] Guilford, J. P. Creative abilities in the arts. *Psychol. Rev*., 10.1037/h0048280 (1957). 10.1037/h0048280 13420286

[13] Cerruti, C. & Schlaug, G. Anodal transcranial direct current stimulation of the prefrontal cortex enhances complex verbal associative thought. *J. Cogn. Neurosci*., 10.1162/jocn.2008.21143 (2009). 10.1162/jocn.2008.21143 PMC3005595 18855556

[14] Mednick, S. The associative basis of the creative process. *Psychol. Rev*., 10.1037/h0048850 (1962). 10.1037/h0048850 14472013

[15] Zmigrod, S., Colzato, L. S. & Hommel, B. Stimulating Creativity: Modulation of Convergent and Divergent Thinking by Transcranial Direct Current Stimulation (tDCS). *Creat. Res. J*., 10.1080/10400419.2015.1087280 (2015).

[16] Bowden, E. M. & Jung-Beeman, M. Normative data for 144 compound remote associate problems. *Behavior Research Methods, Instruments, and Computers*, 10.3758/BF03195543 (2003). 10.3758/bf03195543 14748508

[17] Colombo, B., Bartesaghi, N., Simonelli, L. & Antonietti, A. The combined effects of neurostimulation and priming on creative thinking. A preliminary tDCS study on dorsolateral prefrontal cortex. *Front. Hum. Neurosci*., 10.3389/fnhum.2015.00403 (2015). 10.3389/fnhum.2015.00403 PMC4505103 26236219

[18] Di Bernardi Luft, C., Zioga, I., Banissy, M. J. & Bhattacharya, J. Relaxing learned constraints through cathodal tDCS on the left dorsolateral prefrontal cortex. *Sci. Rep*., 10.1038/s41598-017-03022-2 (2017). 10.1038/s41598-017-03022-2 PMC5462743 28592845

[19] Jacobson, L., Koslowsky, M. & Lavidor, M. TDCS polarity effects in motor and cognitive domains: A meta-analytical review. *Experimental Brain Research*, 10.1007/s00221-011-2891-9 (2012). 10.1007/s00221-011-2891-9 21989847

[20] Lustenberger, C., Boyle, M. R., Foulser, A. A., Mellin, J. M. & Fröhlich, F. Functional role of frontal alpha oscillations in creativity. *Cortex*, 10.1016/j.cortex.2015.03.012 (2015). 10.1016/j.cortex.2015.03.012 PMC4451406 25913062

[21] Grabner, R. H., Krenn, J., Fink, A., Arendasy, M. & Benedek, M. Effects of alpha and gamma transcranial alternating current stimulation (tACS) on verbal creativity and intelligence test performance. *Neuropsychologia*, 10.1016/j.neuropsychologia.2017.10.035 (2017). 10.1016/j.neuropsychologia.2017.10.035 29100950

[22] Terney, D., Chaieb, L., Moliadze, V., Antal, A. & Paulus, W. Increasing Human Brain Excitability by Transcranial High-Frequency Random Noise Stimulation. *J. Neurosci*., 10.1523/JNEUROSCI.4248-08.2008 (2008). 10.1523/JNEUROSCI.4248-08.2008 PMC6671476 19109497

[23] van der Groen, O. & Wenderoth, N. Transcranial Random Noise Stimulation of Visual Cortex: Stochastic Resonance Enhances Central Mechanisms of Perception. *J. Neurosci*., 10.1523/JNEUROSCI.4519-15.2016 (2016). 10.1523/JNEUROSCI.4519-15.2016 PMC6601807 27170126

[24] Schoen, I. & Fromherz, P. Extracellular Stimulation of Mammalian Neurons Through Repetitive Activation of Na+ Channels by Weak Capacitive Currents on a Silicon Chip. *J. Neurophysiol*., 10.1152/jn.90287.2008 (2008). 10.1152/jn.90287.2008 18463183

[25] Snowball, A. *et al*. Long-term enhancement of brain function and cognition using cognitive training and brain stimulation. *Curr. Biol*., 10.1016/j.cub.2013.04.045 (2013). 10.1016/j.cub.2013.04.045 PMC3675670 23684971

[26] Fertonani, A., Pirulli, C. & Miniussi, C. Random Noise Stimulation Improves Neuroplasticity in Perceptual Learning. *J. Neurosci*., 10.1523/jneurosci.2002-11.2011 (2011). 10.1523/JNEUROSCI.2002-11.2011 PMC6703532 22031888

[27] Inukai, Y. *et al*. Comparison of Three Non-Invasive Transcranial Electrical Stimulation Methods for Increasing Cortical Excitability. *Front. Hum. Neurosci*., 10.3389/fnhum.2016.00668 (2016). 10.3389/fnhum.2016.00668 PMC5186778 28082887

[28] Vanneste, S., Fregni, F. & De Ridder, D. Head-to-head comparison of transcranial random noise stimulation, transcranial AC stimulation, and transcranial DC stimulation for tinnitus. *Front. Psychiatry*, 10.3389/fpsyt.2013.00158 (2013). 10.3389/fpsyt.2013.00158 PMC3866637 24391599

[29] Bieck, S. M., Artemenko, C., Moeller, K. & Klein, E. Low to no effect: Application of tRNS during two-digit addition. *Front. Neurosci*., 10.3389/fnins.2018.00176 (2018). 10.3389/fnins.2018.00176 PMC5895770 29674948

[30] Looi, C. Y. *et al*. Transcranial random noise stimulation and cognitive training to improve learning and cognition of the atypically developing brain: A pilot study. *Sci. Rep*., 10.1038/s41598-017-04649-x (2017). 10.1038/s41598-017-04649-x PMC5498607 28680099

[31] Antal, A. *et al*. Brain-derived neurotrophic factor (BDNF) gene polymorphisms shape cortical plasticity in humans. *Brain Stimulation*, 10.1016/j.brs.2009.12.003 (2010). 10.1016/j.brs.2009.12.003 20965453

[32] Simonsmeier, B. A., Grabner, R. H., Hein, J., Krenz, U. & Schneider, M. Electrical brain stimulation (tES) improves learning more than performance: A meta-analysis. *Neuroscience and Biobehavioral Reviews*, 10.1016/j.neubiorev.2017.11.001 (2018). 10.1016/j.neubiorev.2017.11.001 29128578

[33] Ruggiero, F., Lavazza, A., Vergari, M., Priori, A. & Ferrucci, R. Transcranial Direct Current Stimulation of the Left Temporal Lobe Modulates Insight. *Creat. Res. J*., 10.1080/10400419.2018.1446817 (2018).

[34] Aihara, T., Ogawa, T., Shimokawa, T. & Yamashita, O. Anodal transcranial direct current stimulation of the right anterior temporal lobe did not significantly affect verbal insight. *PLoS One*, 10.1371/journal.pone.0184749 (2017). 10.1371/journal.pone.0184749 PMC5597234 28902872

[35] Ivancovsky, T., Kurman, J., Morio, H. & Shamay-Tsoory, S. Transcranial direct current stimulation (tDCS) targeting the left inferior frontal gyrus: Effects on creativity across cultures. *Social Neuroscience*, 10.1080/17470919.2018.1464505 (2018). 10.1080/17470919.2018.1464505 29641936

[36] Zmigrod, S., Colzato, L. S. & Hommel Lorenza, S. ORCID: http://orcid.org/0000-0003-1735-1280, B. A. I.-O. http://orcid. org/Colzat. Stimulating creativity: Modulation of convergent and divergent thinking by transcranial direct current stimulation (tDCS). *Creativity Research Journal* (2015).

[37] Ghanavati, E., Nejati, V. & Salehinejad, M. A. Transcranial Direct Current Stimulation over the Posterior Parietal Cortex (PPC) Enhances Figural Fluency: Implications for Creative Cognition. *J. Cogn. Enhanc*., 10.1007/s41465-017-0059-7 (2018).

[38] Badzakova-Trajkov, G., Häberling, I. S. & Corballis, M. C. Magical ideation, creativity, handedness, and cerebral asymmetries: A combined behavioural and fMRI study. *Neuropsychologia*, 10.1016/j.neuropsychologia.2011.06.016 (2011). 10.1016/j.neuropsychologia.2011.06.016 21722656

[39] Oldfield, R. C. The assessment and analysis of handedness: The Edinburgh inventory. *Neuropsychologia*, 10.1016/0028-3932(71)90067-4 (1971). 10.1016/0028-3932(71)90067-4 5146491

[40] Schretlen, D. J. & Vannorsdall, T. D. *Calibrated Ideational Fluency Assessment (CIFA) Professional Manual* (Psychological Assessment Resources Inc., 2010).

[41] Brunyé, T. T. *et al*. Increasing breadth of semantic associations with left frontopolar direct current brain stimulation: A role for individual differences. *Neuroreport*, 10.1097/WNR.0000000000000348 (2015). 10.1097/WNR.0000000000000348 25714417

[42] Steele, L. M., Johnson, G. & Medeiros, K. E. Looking beyond the generation of creative ideas: Confidence in evaluating ideas predicts creative outcomes. *Pers. Individ. Dif*., 10.1016/j.paid.2017.12.028 (2018).

[43] Kim, K. H. Can we trust creativity tests? A review of the Torrance Tests of Creative Thinking (TTCT). *Creat. Res. J*., 10.1207/s15326934crj1801_2 (2006).

[44] Torrance, E. P. Torrance Tests of Creative Thinking. *J. Educ. Meas*., 10.1037/t05532-000 (1967).

[45] Miranda, P. C., Mekonnen, A., Salvador, R. & Ruffini, G. The electric field in the cortex during transcranial current stimulation. *Neuroimage*, 10.1016/j.neuroimage.2012.12.034 (2013). 10.1016/j.neuroimage.2012.12.034 23274187

[46] Faul, F., Erdfelder, E., Lang, A.-G. & Buchner, A. G*Power 3: A flexible statistical power analysis program for the social, behavioral, and biomedical sciences. *Behav. Res. Methods*, 10.3758/BF03193146 (2007). 10.3758/bf03193146 17695343

[47] Shapiro, S. S., Wilk, M. B. & Chen, H. J. A Comparative Study of Various Tests for Normality. *J. Am. Stat. Assoc*., 10.1080/01621459.1968.10480932 (1968).

[48] Efron, B. & Tibshirani, R. J. *An Introd. to bootstrap* (Chapman and Hall; New York, NY: 1993).

[49] IBM Corp. Released. IBM SPSS Statistics for Windows, Version 23.0. 2015 (2015).

